# British Columbia Children’s Hospital Compass Program: Extending mental health supports for rural Northern communities

**DOI:** 10.1371/journal.pone.0340735

**Published:** 2026-05-14

**Authors:** Vivian W. L. Tsang, Annie Kim, Tiffany Y. X. Wu, Lynne Hill, Matt Burkey, Megan Crawford, Erica Koopmans, Chantelle Wilson, Roberto Sassi

**Affiliations:** 1 Department of Psychiatry, Faculty of Medicine, University of British Columbia, Detwiller Pavilion, Wesbrook Mall, Vancouver, British Columbia, Canada; 2 Undergraduate Medical Program, Faculty of Medicine, University of British Columbia, Vancouver, British Columbia, Canada; 3 Faculty of Science, University of British Columbia, University Blvd, Vancouver, British Columbia, Canada; 4 Compass program, Child Psychiatry, BC Children’s Hospital, Vancouver, British Columbia, Canada; 5 Northern Health Authority, Prince George, British Columbia, Canada; 6 Child Health BC, Provincial Health Services Authority, Vancouver, British Columbia, Canada; 7 School of Health Sciences, Faculty of Health and Human Services, University of Northern British Columbia, University Way, Prince George, British Columbia, Canada; 8 Child and Youth Service Network, Prince George, British Columbia, Canada; University of Toronto, CANADA

## Abstract

**Purpose:**

This article evaluates the impact of the Compass Program over the last six years, with a focus on access to mental health services in Northern British Columbia (BC) and Indigenous communities within the region.

**Methods:**

Compass program data from September 2018 to April 2024 were analyzed. Demographic and clinical characteristics were compared between Northern and other BC regions. Quantitative variables were summarized using descriptive statistics.

**Findings:**

Northern BC accounts for 21.4% of total Compass encounters. Indigenous youth in this region are particularly underserved, and community providers are more likely to request specialized virtual consultations at Compass than other regions. Anxiety was the most common presenting concern across all regions. Northern BC represented 46% of all Indigenous consultations and showed a steady increase in Indigenous-related calls—from 18% in 2018 to 41% in 2024. Among direct consults in Northern BC, 55% involved Indigenous patients and medication questions were the most common reasons for calling.

**Conclusions:**

The study highlights the distinct mental health challenges in Northern BC compared to other regions of the province, suggesting the unique socioeconomic and geographical factors contributing to different mental health issues. Further research should be done to explore the impact of specialized child and youth mental health and substance use provider-to-provider consultations in other similarly underserved areas in Canada. The Compass program at BC Children’s Hospital provides a model that tries to navigate mental health disparities of Northern BC, where there is high demand for mental health services. **Clinical trials registry number:** H23-03251-A001.

## Introduction

British Columbians who live in rural Northern communities face unique challenges accessing mental health services [1,2]. Northern Health is the governmental authority responsible for the health of the population in Northern British Columbia (BC), covering the largest geographic health region in the province, spanning North of Quesnel to the Yukon border and from the Alberta border West to Haida Gwaii, which covers 64.0% of the provincial land base [1]. Northern Health has a population of around 290,000, representing about 5% of the total province of BC [2]. It comprises mostly sparsely populated rural communities, making access to mental health support difficult. Around 20% of the population in the Northern Health region identifies as Indigenous, which is the highest proportion in the province [2]. The Northern Health Authority has dedicated significant resources to improve access to mental health and substance use care across their region, but multiple challenges persist, including difficulties in recruiting and retaining qualified personnel across a vast geographic area. As a consequence, there are still significant limitations in access to secondary and tertiary child and youth psychiatric services for youth living in Northern BC, leaving primary care and community services in the area with the responsibility to manage complex presentations that would have been referred to secondary or tertiary specialized mental health programs in other parts of the province.

This issue is even more acute for Indigenous populations, who face additional barriers accessing culturally appropriate mental health services in these communities. Jurisdictional and fiscal challenges, as outlined in Jordan’s Principle, a child-first principle to ensure First Nations children receive appropriate care, often result in inequitable distribution of resources and services [3]. Furthermore, educational disparities in rural areas, such as high turnover of school staff, particularly in Indigenous communities, exacerbates this issue. The lack of education surrounding social emotional learning and health education contribute to limited support for youth [4]. These challenges compound with multigenerational trauma from residential and day schools, leaving many youths without adequate access to needed mental health services [5]. The Western medical model has imposed historical and ongoing systemic harm and barriers that continue to significantly impact Indigenous youth [6]. Despite multi-generational trauma, cultural loss, and inequitable access to care, Indigenous youth and communities rightfully deserve adequate and culturally safe mental health care.

There has been increasing focus on mental health and addictions strategies for Northern regions, given their specific geographic and cultural differences [7,8]. In areas with high rates of youth mental illness or addiction, early intervention programs targeted at youth have been implemented [7]. However, as of 2018, child-psychiatry specific telepsychiatry programs aiming to ensure timely support to clinicians in Northern Health were not widely available.

The BC Children’s Hospital Compass program was developed to fill this gap. Compass is a first of its kind province-wide initiative in Canada based on similar programs in the United States [8] providing access to specialized child and youth mental health and substance use expertise to community clinicians, physicians, nurse practitioners, school counselors and Indigenous Elders across the province who are caring for children and youth (0–25) living with mental health and substance use concerns [9]. The multidisciplinary team at Compass includes child and adolescent psychiatrists, psychologists, nurse clinicians, social workers, youth and family counselors, registered clinical counselors and Indigenous liaisons. Providers from across the province can call into Compass where an interdisciplinary meeting will be facilitated to a child psychiatrist for consultation. These are termed indirect consultations. This model improves access to specialist advice and treatment plans, while also building mental health management capacity among local providers. Of note, through a contract between BC Children’s Hospital and the Government of British Columbia, Compass also provides direct consultations for a small number of patients living within the Northern Health region, helping to address health human resource gaps in the region and enhance local capacity through access to specialist expertise [10].

The purpose of this retrospective study is to characterize the results of the Compass program’s operations in Northern Health over its first five years of operation and to evaluate the resulting utilization patterns in these rural or remote communities, with a specific focus on Indigenous populations. By examining the demographics and services accessed, this study aims to provide insight into the reach of programs like Compass and to identify areas of improvement to better serve rural and remote populations. The results of this study have the potential to derive important insights into the unmet needs and inform ongoing and new psychiatric access programs for children and adolescents in Northern BC as well as other northern and rural or remote communities.

## Methods

### Study design and setting

Northern Health providers have been engaged in Compass operations since its launch in September of 2018. This study captures data from inception to April 2024 with population data sourced from the 2016 Census by Statistics Canada [5].

### Data sources

Clinical operations of the Compass program have already been well documented in previous studies [11]. The present study focuses on data from the Compass RedCap database which includes patient demographics, provider demographics, presenting concerns, consultation questions, and outcomes from follow-up care. Data were inputted into RedCap by a mental health clinician during each encounter. Data was initially collected for quality improvement purposes with secondary use of data approved through ethics from the University of British Columbia Research Ethics Board (#H23-03251-A001).

### Study population and encounter definitions

This study focuses on encounters that are geographically associated with Northern Health only. This includes indirect consultations provided for Northern Health providers as well as direct consultations with patients which is a service between Compass and Northern Health to address the paucity of specialists in the region. An encounter would include any length of consultation provided between the Compass team and a provider. These encounters ranged in length between fifteen minutes to ninety minutes. Encounters with a patient can include multiple calls, such as an initial call as well as follow-up calls for the same patient case. However, a patient could have more than one encounter in the RedCap database if multiple providers called regarding the same patient, or if the provider called for a different issue or started a new case unrelated to the previous case. Encounters or calls where the variables of interest were missing were excluded from the corresponding visualizations.

### Data analysis

All analyses were conducted using R statistical software (Version 4.2.1), and data visualizations were generated using the ggplot2 package (Version 3.4.2). Quantitative descriptive statistics were calculated to summarize demographic, geographic, and consultation characteristics. These included frequencies and proportions for categorical variables, as well as measures of central tendency (means and medians) and variability (standard errors and confidence intervals) where appropriate.

Patient-level analyses were stratified by key variables of interest, including age, gender, reasons for consultation, presenting concerns, and Indigenous identity. Age was analyzed as a continuous variable and displayed by single-year increments to illustrate consultation patterns across the 0–25 age range. Gender was analyzed categorically; to ensure sufficient sample size and protect interpretability, gender identities other than male or female were collapsed into an “other” category for comparative analyses. Indigenous identity was analyzed as a categorical variable, with analyses excluding encounters where Indigenous status was recorded as “not asked” or “provider did not know,” unless otherwise specified.

To facilitate meaningful comparisons and ensure adequate cell sizes, select subcategories were collapsed prior to analysis. This approach was applied consistently across Northern and non-Northern regions to preserve comparability. Missing data were handled using complete-case analysis on a per-analysis basis. That is, encounters with missing values for a given variable were excluded only from analyses involving that variable, rather than from the entire dataset. The number of excluded encounters therefore varied across analyses and figures.

Comparative analyses between Northern Health and other regions of British Columbia were descriptive and visual rather than inferential, reflecting the study’s exploratory objectives. Where statistical testing was conducted, a two-sided significance threshold of p < 0.05 was used. No formal adjustments were made for multiple comparisons, as analyses were hypothesis-generating rather than confirmatory. Results were visualized using composite bar graphs, pie charts, and line plots to illustrate distributions and trends across regions, demographic groups, and time. For select figures (including Figs 2 and 5), the y-axis scales differ across panels. These non-equivalent scales were intentionally used to better convey proportional differences within each subgroup and region, rather than to facilitate direct visual comparison of absolute values across panels. All analyses were conducted using reproducible R scripts, and figures were generated directly from the analytic datasets to ensure consistency between reported values and visualizations.

**Fig 1:**
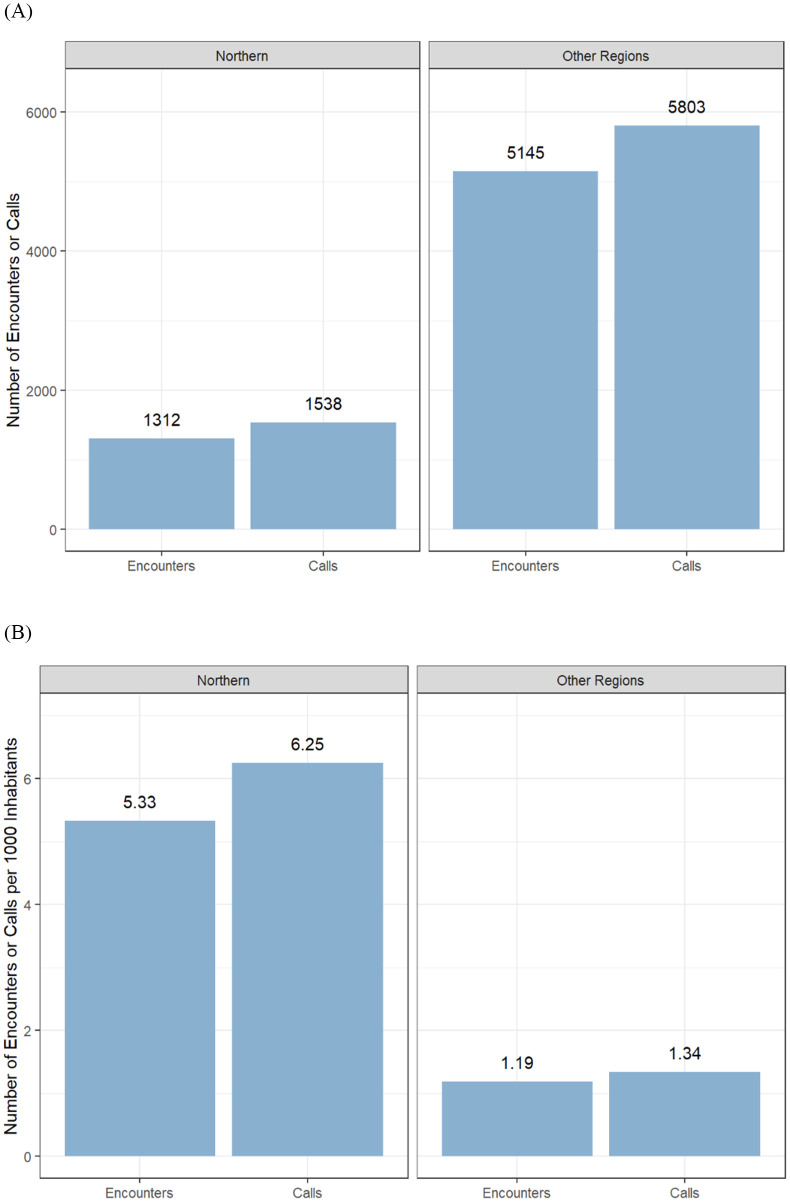
Compass program encounters and calls by health region in British Columbia. **(A)** Absolute number of encounters and calls from Northern Health compared with other provincial health regions. **(B)** Relative proportion of encounters and calls from Northern Health compared with other provincial health regions.

**Fig 2:**
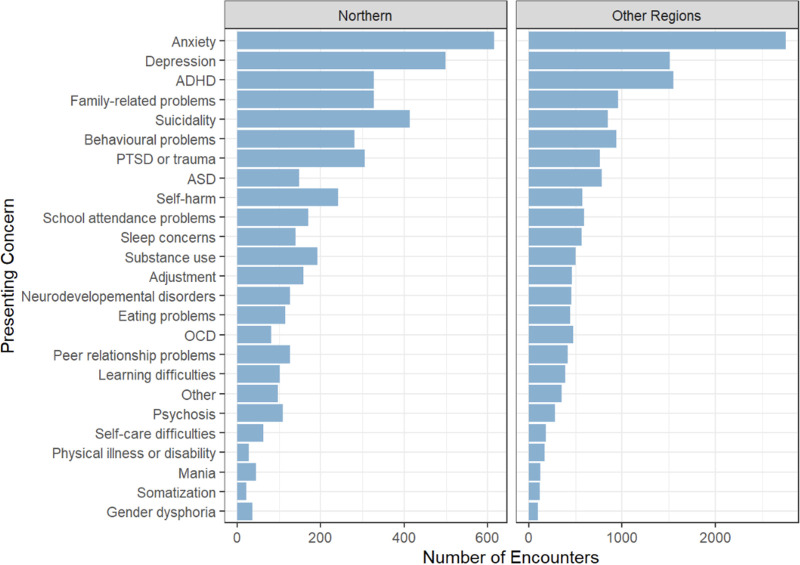
Presenting concerns in Northern Health compared to other regions in British Columbia.

**Fig 3:**
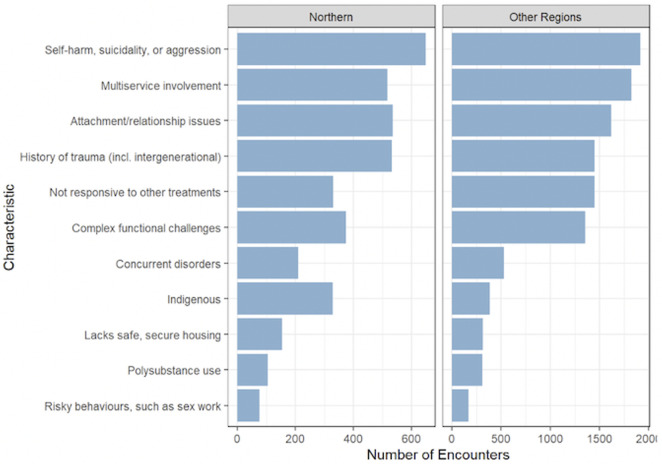
Ministry of health-identified important concerns in Northern vs. other regions of British Columbia.

**Fig 4:**
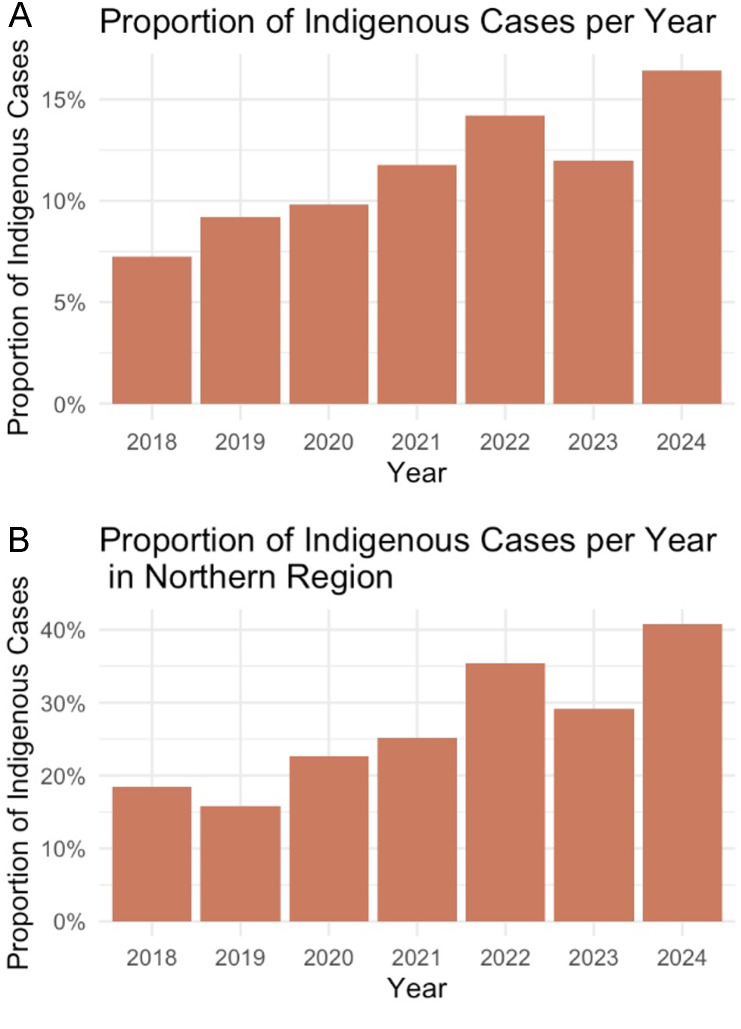
Proportion of Indigenous cases received per year. **(A)** Annual proportion of encounters across all regions of BC. **(B)** Annual proportion of encounters in Northern BC.

## Results

Encounters from Northern Health (*n* = 1312) make up 21.4% of the raw percentage of total Compass encounters. However, when accounting for population differences, the number of encounters per 1000 inhabitants between the ages of 0–24 soars close to five times that of other regions in BC (Fig 1, S1-S2 Fig).

The number of encounters by patient gender between Northern Health and other regions in BC are similar with a higher number of females in both groups (55% encounters in Northern Health; 50% encounters in other regions). There are no significant differences between other gender-identifying individuals in both groups. Ages of patients discussed in Northern Health encounters ranged between 1–25 with the average age. Both Northern Health and other regions have an approximate normal distribution with a negative skew. Both show similar trends with patients at age 15 (15% for Northern Health; 13.5% for other regions) having the highest number of encounters followed by patients who are age 16. There is a sharp decline seen in the number of encounters of patients after age 18 which is reflected in other regions as well.

Trends in reasons for calling are proportionally similar between Northern Health and other regions, with **medication questions** comprising the highest frequency of calls, followed by **resource coordination** or **community access and therapy/behavioral interventions**. However, there are slight differences in presenting concerns by region (Fig 2, S3–S5 Figs). While **anxiety** continues to be the highest presenting issue by frequency, the next concerns in Northern Health are **depression** at 499 encounters, followed by **suicidality** at 413 encounters. This is then followed by attention deficit hyperactivity disorder (ADHD) and **family-related problems** each at 327 encounters. post-traumatic stress disorder (PTSD) or **trauma** encounters follow closely at 306 encounters and then followed by **behavioral problems**. In other regions in BC, suicidality (fifth) and PTSD or trauma-related issues (eighth) are further down the list of presenting concerns.

The number of providers accessing Compass for cases related to self-harm, suicidality, or aggression is similar between regions with 17.0% of total encounters in Northern Health and 16.9% in other regions. This is followed by attachment or relationship issues in Northern Health at 14.0% of total encounters and multiservice involvement for other regions at 16.1% of total encounters.

### Impact on indigenous patients

To determine the impact of Compass on Indigenous communities overall, we analyzed the percentage of consultations for Indigenous patients Compass receives across all regions on a yearly basis. Generally, there is an increasing pattern of calls from providers about Indigenous children/youth. The percentage of consultations ranges between 7.3% in 2018 to 16.4% in 2024.

More importantly, there is an increasing pattern of calls from providers about Indigenous children/youth in the Northern region, indicating an upwards trajectory in the impact of Compass on healthcare in Indigenous children/youth (Fig 3). The number of cases was lowest in 2019 at 15.9% and highest in 2024 at 40.8%. Generally, there is an increasing pattern of calls from providers about Indigenous children/youth, though there is a slight drop in 2023 (from 35.3% in 2022 to 29.2% in 2023). In addition, the number of consultations to providers regarding Indigenous children and youth is the highest in the Northern region, comprising 46% of total Indigenous consults (Fig 3). Other regions, except Yukon, have a similar absolute number of consults, ranging from 53 to 72. This graph excludes the categories “not asked” and “provider did not know.”

We were also interested in investigating the complexity of cases in the Northern region versus other regions. To evaluate this, we looked at the number of calls it takes to resolve a case. A chi-squared test was completed. The total number of observations was: Northern BC = 1312, other regions of BC = 5145. The percentage of follow-ups was: Northern BC = 10.36%, other regions of BC = 11.78%. Our results do not indicate a significant difference (χ2(1, N = 6457) = 2.05, p = 0.15) in the percentage of cases with repeat calls, with roughly 10% in the Northern region and 12% for all other regions.

The percentage of female patients remains the same, both when looking at Indigenous cases specifically and the total gender distribution (42.9% in the total gender distribution compared to 43% when looking specifically at Indigenous cases). A lower percentage of males were found to be accessing Compass among Indigenous populations than in the overall distribution but was overall not significant (34.8% of Indigenous cases were from male patients, while 37.6% of total Compass cases were from male patients (χ2(1, N = 598) = 0.003, p = 0.96)). However, a higher percentage of Indigenous patients reported “Other” genders (“Other” including “Cisgender”,”Transgender”,”Non-Binary”,”Gender Creative or Gender Variant”,”Gender Nonconforming”,”Agender”,”Other”), with 11.7% reporting “Other” genders when looking specifically at Indigenous Compass participants compared to 6.8% in the total gender distribution. “Unknown” classifications account for missing data as well as situations where providers that called Compass did not identify the gender of the youth they were calling about.

### Direct call services

As noted, a unique service that the BC Children’s Hospital Compass program provides for a small number of patients living in the Northern Health authority is direct consultations. In these calls, a Compass child psychiatrist would see a patient over Zoom and conduct a complete psychiatric interview with a psychiatric assessment and plan. They then usually engage in a series of follow-up calls with local providers in the Northern Health region to equip health care providers in the area with the necessary knowledge and resources for ongoing management of their patient (S4 Fig).

To determine the types of patients that use the direct call services, we conducted an analysis of direct assessments across multiple demographic factors. Our results indicate that 55% of direct consults were made up of Indigenous cases and 43% were non-Indigenous cases (Fig 4).

Secondly, we examined the percentage of direct consults across different gender categories. There were minimal differences between females and males with only a slightly higher percentage of direct consults for females at 40%.

Next, we explored potential differences in age distribution among individuals utilizing the direct consult service. The overall trend is similar with teenagers aged 15 receiving the highest number of consults in direct consults (N = 12) and teenagers aged 14 in indirect consults (N = 590).

**Fig 5:**
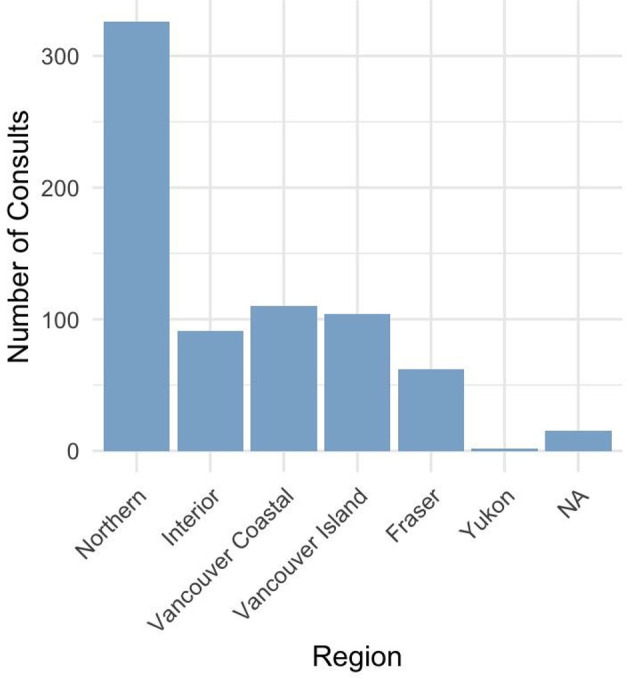
Total number of consultations involving Indigenous patients by region. This graph excludes the categories “not asked” and “provider did not know”.

**Fig 6:**
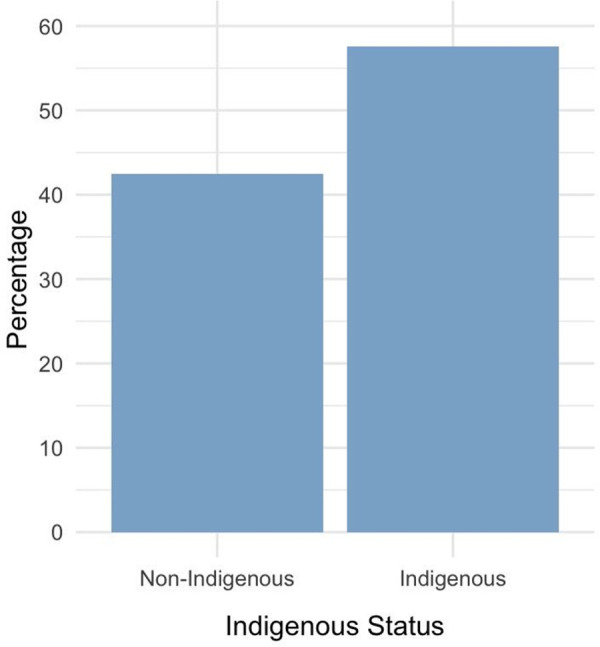
Percentage of direct consults by Indigenous status.

**Fig 7:**
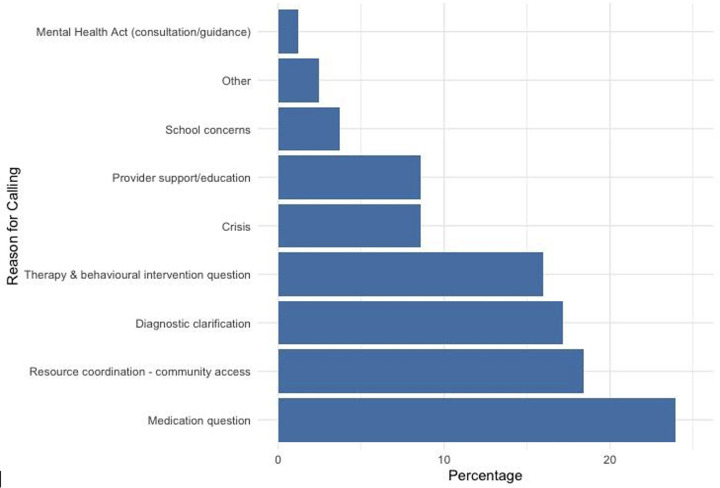
Percentage distribution of reasons for calling in direct consults.

**Fig 8:**
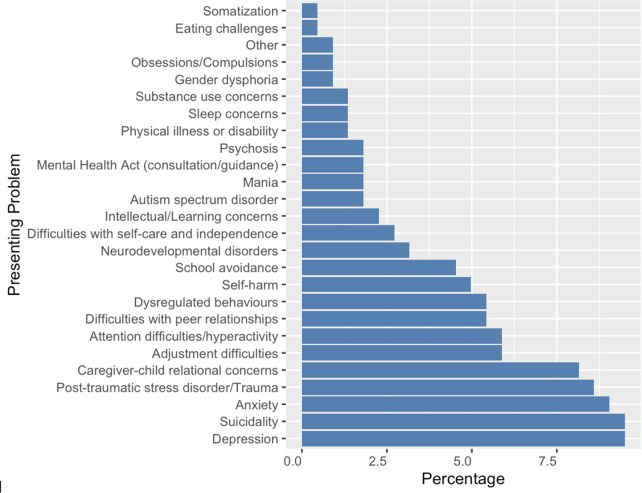
Percentage distribution of presenting problems in direct consults.

We also analyzed the reasons for calling across direct consults to better understand the nature of services provided. Our analysis revealed that medication questions were the most common reasons for calling followed by resource coordination for community access (Figs 5–7). Depression, suicidality, and anxiety are consistently ranked among the top three most common presenting problems in direct consults (Fig 8).

We were also interested in investigating the complexity of cases for direct consults. To evaluate this, we looked at the number of calls it takes to resolve a case. The data revealed that the majority of both direct consults (approximately 70%) and indirect consults (approximately 88%) are resolved in a single call, with no follow-up required. For cases requiring follow-up, the data indicate that it is rare for cases to exceed two calls in both consult types.

## Discussion

The BC Children’s Hospital Compass Program has expanded substantially since its inception, supporting over 2,336 providers and managing more than 5,709 cases during its first five years of operation [11]. This program is uniquely positioned to address longstanding gaps in access to specialized child and youth mental health care in geographically large, sparsely populated regions. While child psychiatry access programs have been evaluated in other jurisdictions, few studies have examined their impact within northern, rural, or Indigenous contexts [12]. Within British Columbia, our findings demonstrate that utilization of Compass services by Northern Health providers is disproportionately high relative to population size, highlighting the significant unmet need for child and adolescent psychiatric expertise in rural and remote communities.

The markedly higher per-capita utilization of Compass in Northern BC reflects both limited access to specialist services and the reliance of community-based providers on virtual consultation models to manage increasingly complex presentations. In many Northern communities, primary care providers, community mental health clinicians, and school-based professionals are required to manage cases that would typically be referred to secondary or tertiary services in urban settings. Geographic isolation, workforce shortages, and challenges with recruitment and retention further constrain access to in-person psychiatric care [1,6,7]. Within this context, Compass functions as a critical extension of the mental health system, enabling timely access to interdisciplinary expertise and supporting local providers in delivering care that might otherwise be unavailable.

Patterns in presenting concerns further illustrate the distinct mental health landscape of Northern BC. Although anxiety was the most common presenting concern across all regions, Northern Health encounters demonstrated relatively higher frequencies of depression, suicidality, and trauma-related concerns compared to other regions. These findings are consistent with literature describing elevated exposure to social and structural stressors in rural and remote communities, including poverty, housing instability, limited educational resources, and higher rates of violence [13]. The prominence of suicidality as a leading concern in Northern BC underscores the importance of rapid access to specialist consultation for risk assessment and management, particularly in communities where emergency and inpatient psychiatric resources are limited.

Despite these differences in clinical presentation, the proportion of cases requiring repeat consultations did not differ significantly between Northern Health and other regions. This finding suggests that a single Compass consultation is often sufficient to support local providers, even when managing complex or high-acuity cases. From a system-level perspective, this supports the efficiency of the Compass model and its ability to enhance provider confidence and decision-making without generating excessive follow-up demand. In rural settings where provider isolation is common, timely access to specialist input may reduce unnecessary referrals, support continuity of care, and strengthen local capacity to manage mental health concerns [14].

The findings related to Indigenous youth are particularly salient. Indigenous patients accounted for nearly half of all Indigenous-related Compass consultations province-wide, with the proportion of Indigenous cases in Northern Health increasing steadily over time. This trend likely reflects both the demographic composition of Northern BC and the significant service gaps faced by Indigenous communities in the region. Indigenous youth were also overrepresented in direct psychiatric consultations, suggesting that Compass is being used to address critical shortages in specialized care for Indigenous patients. However, increased utilization should not be interpreted as an indication that existing needs are being fully met. Rather, it underscores the absence of accessible, culturally appropriate mental health services in many Indigenous communities and the reliance on virtual consultation as a substitute for locally available care [15–19].

Indigenous patients continue to experience systemic barriers within the mental health system, including racism, discrimination, underfunding, and services that are misaligned with cultural values and healing practices [6,16,17]. Historical and ongoing impacts of colonialism have contributed to mistrust of healthcare institutions, further limiting access to care [20,21]. While Compass cannot resolve these structural inequities, the program has made efforts to promote cultural humility through the inclusion of Indigenous care coordinators and social workers. These initiatives may contribute to safer and more responsive care pathways; however, they must be accompanied by sustained investment in Indigenous-led, community-based mental wellness services that reflect local knowledge systems and priorities.

The predominance of medication-related questions across both direct and indirect consultations highlights another important implication. Providers in Northern BC are frequently navigating complex pharmacologic decisions in contexts where access to psychiatric oversight is limited. While Compass supports safer prescribing and clinical decision-making, medication-focused interventions alone are insufficient to address the broader social, emotional, spiritual, and cultural determinants of mental health, particularly for Indigenous youth. Comprehensive mental wellness requires integrated approaches that include psychosocial supports, community resources, and culturally grounded care [17,18].

Taken together, these findings have broader implications for mental health system planning in rural and remote regions across Canada. The Compass program demonstrates how virtual consultation models can enhance access to specialist expertise, support local providers, and partially mitigate geographic inequities. However, such programs should be viewed as complementary to, rather than replacements for, long-term investments in workforce development, culturally safe services, and community-driven care. Without parallel efforts to address these systemic factors, virtual access programs risk becoming stopgap solutions rather than mechanisms for sustainable change.

In summary, this study highlights the critical role of the Compass program in supporting child and youth mental health care in Northern British Columbia, particularly for Indigenous communities. The disproportionately high utilization of Compass services reflects both significant unmet need and the program’s importance as a clinical and capacity-building resource. As other jurisdictions seek to improve access to mental health care in underserved regions, Compass offers a promising model—one that must continue to evolve in partnership with communities to promote equity, cultural safety, and meaningful access to care.

### Strengths and limitations

This study draws on a large, longitudinal dataset spanning more than five years of Compass program operations, allowing for a comprehensive assessment of utilization patterns over time. The study also places emphasis on disparities in the Northern Health region, an underserved region in British Columbia as well as on Indigenous youth, an underserved demographic in the province. The use of routinely collected program data enhances the ecological validity of the findings and reflects real-world clinical practice across diverse rural and remote settings and the use of population-adjusted encounter rates strengthens the interpretation of regional differences by accounting for substantial population size disparities across British Columbia.

Several limitations should also be considered when interpreting the results. First, the study relies on retrospective administrative data collected primarily for quality improvement purposes, which limits the availability of certain variables and may introduce inconsistencies in data entry. Encounters rather than unique patients were used as the unit of analysis, which may overrepresent individuals with multiple consultations and limit the ability to draw conclusions about patient-level outcomes. The analyses were largely descriptive and exploratory in nature, and causal inferences cannot be drawn regarding the impact of Compass on clinical outcomes. This study also did not have the opportunity to involve youth in consultation which would be an important next step to future work in this area [22,23].

### Conclusions

The disproportionately high utilization of Compass services in the Northern Health region, when adjusted for population, underscores the critical need for specialized mental health and psychiatric support for children and youth in rural and remote areas. The data also reveals that Indigenous youth are among the most frequent users of Compass consultations, especially the direct consult services tailored to address the region’s health human resource gaps. While the program has made strides in promoting cultural humility and responsiveness through the inclusion of Indigenous care coordinators and social workers there remains a need for continued investment in culturally safe, community driven mental health services in BC. The findings highlight the importance of sustained collaboration, not only to address historical and systemic inequities, but also to deliver virtual mental wellness support rooted in Indigenous knowledge and traditions. Compass serves as a promising model for other underserved regions, demonstrating how virtual consultation services can enhance local capacity and promote equity in mental health service access.

## Supporting information

S1 Fig Patient encounters by demographic characteristics in Northern vs. other regions of British Columbia. (A) Distribution of encounters by patient gender. (B) Distribution of encounters by patient age.
(DOCX)

S2 Fig Gender distribution of patient encounters received by Compass. (A) Overall gender distribution across all encounters. (B) Gender distribution among patients who self-identified as Indigenous. “Other” includes respondents identifying as Cisgender, Transgender, Non-Binary, Gender Creative or Gender Variant, Gender Nonconforming, Agender, or Other.
(DOCX)

S3 Fig Percentage of cases with repeat calls in Northern British Columbia versus other regions. Total number of encounters in Northern BC = 1,312 and other BC regions = 5,145.
(DOCX)

S4 Fig Percentage of direct consults by patient gender in Northern British Columbia. (DOCX)

S5 Fig Age distribution of patient encounters by type of consult. (A) Percentage of direct consults. (B) Percentage of indirect consults.
(DOCX)

S6 Fig Number of repeat calls to resolve a case by type of consult. (A) Percentage of direct consults. (B) Percentage of indirect consults.
(DOCX)
