# Supplementary material for: British Columbia Children’s Hospital Compass Program: Extending mental health supports for rural Northern communities
Source: PLoS One. 2026 May 14;21(5):e0340735. doi: 10.1371/journal.pone.0340735 (PMC13175457; doi:10.1371/journal.pone.0340735)
Supplement: S1 Fig — (A) Distribution of encounters by patient gender. (B) Distribution of encounters by patient age. (DOCX) [file pone.0340735.s001.docx]

**(A)**


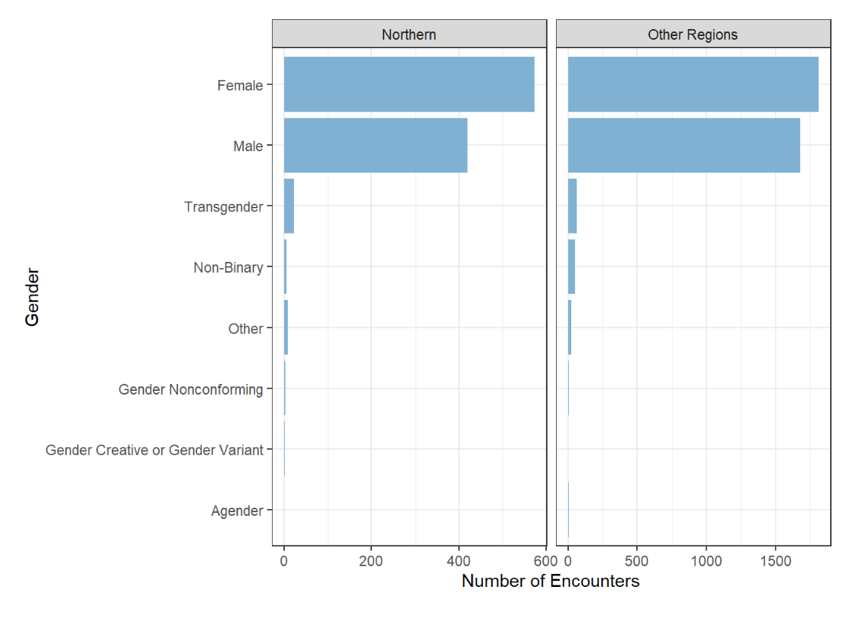


**(B)**

**
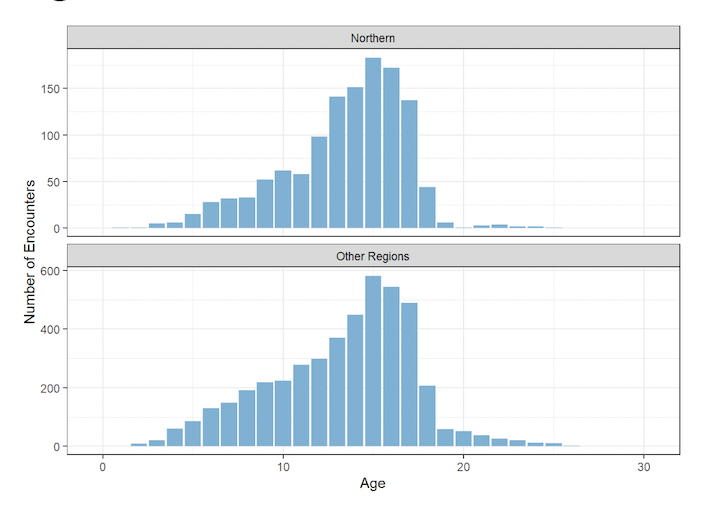
**

**Sup Fig 1. Patient encounters by demographic characteristics in Northern vs. other regions of British Columbia. (**A) Distribution of encounters by patient gender. (B) Distribution of encounters by patient age.
