# Supplementary material for: British Columbia Children’s Hospital Compass Program: Extending mental health supports for rural Northern communities
Source: PLoS One. 2026 May 14;21(5):e0340735. doi: 10.1371/journal.pone.0340735 (PMC13175457; doi:10.1371/journal.pone.0340735)
Supplement: S2 Fig — (A) Overall gender distribution across all encounters. (B) Gender distribution among patients who self-identified as Indigenous. “Other” includes respondents identifying as Cisgender, Transgender, Non-Binary, Gender Creative or Gender Variant, Gender Nonconforming, Agender, or Other. (DOCX) [file pone.0340735.s002.docx]

(A)


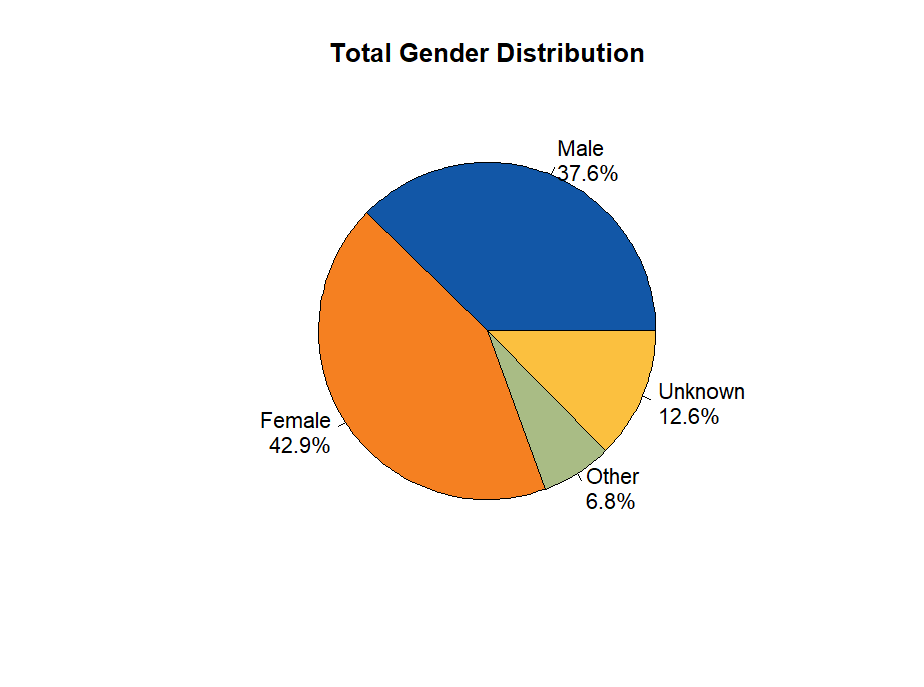


(B)


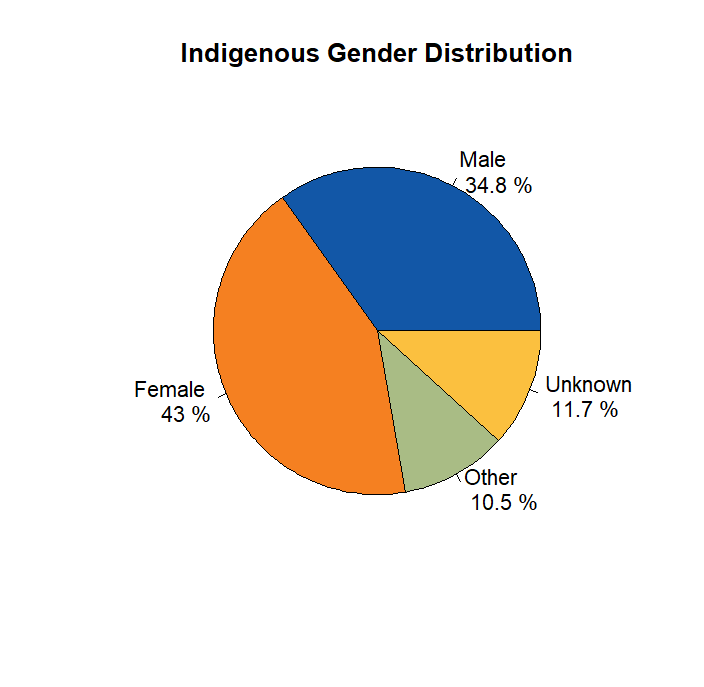


**Sup Fig 2. Gender distribution of patient encounters received by Compass.** (A) Overall gender distribution across all encounters. (B) Gender distribution among patients who self-identified as Indigenous. “Other” includes respondents identifying as Cisgender, Transgender, Non-Binary, Gender Creative or Gender Variant, Gender Nonconforming, Agender, or Other.
