# Supplementary material for: British Columbia Children’s Hospital Compass Program: Extending mental health supports for rural Northern communities
Source: PLoS One. 2026 May 14;21(5):e0340735. doi: 10.1371/journal.pone.0340735 (PMC13175457; doi:10.1371/journal.pone.0340735)
Supplement: S3 Fig — Total number of encounters in Northern BC = 1,312 and other BC regions = 5,145. (DOCX) [file pone.0340735.s003.docx]

**
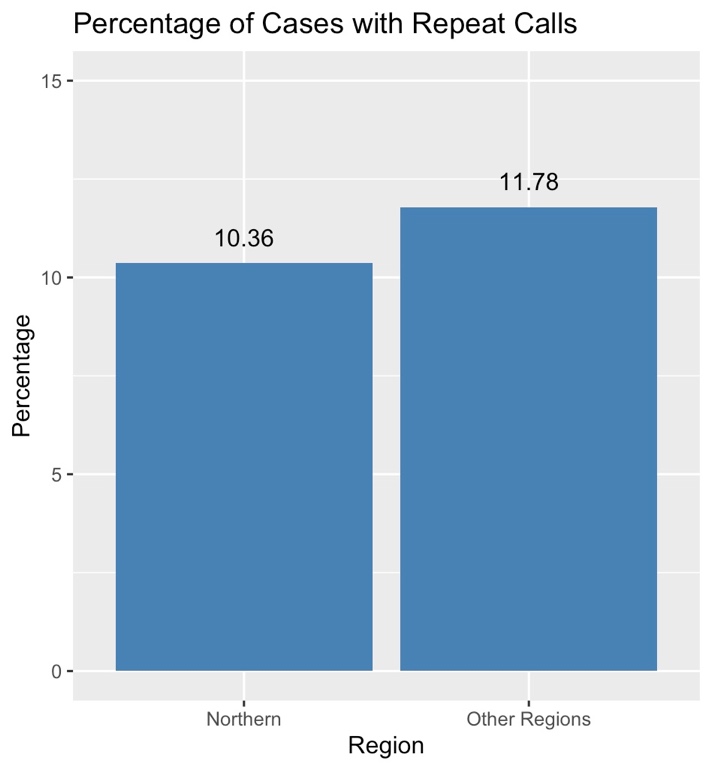
**

**Sup Fig 3. Percentage of cases with repeat calls in Northern British Columbia versus other regions.** Total number of encounters in Northern BC = 1,312 and other BC regions = 5,145.
