# Supplementary figures and images for: British Columbia Children’s Hospital Compass Program: Extending mental health supports for rural Northern communities
Source: PLoS One. 2026 May 14;21(5):e0340735. doi: 10.1371/journal.pone.0340735 (PMC13175457; doi:10.1371/journal.pone.0340735)

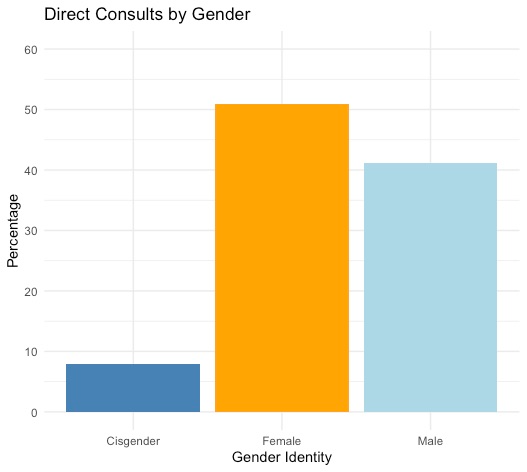


**Sup Fig 4. Percentage of direct consults by patient gender in Northern British Columbia.**

Supplement: S4 Fig — (DOCX) [file pone.0340735.s004.docx]
