# Supplementary material for: British Columbia Children’s Hospital Compass Program: Extending mental health supports for rural Northern communities
Source: PLoS One. 2026 May 14;21(5):e0340735. doi: 10.1371/journal.pone.0340735 (PMC13175457; doi:10.1371/journal.pone.0340735)
Supplement: S5 Fig — (A) Percentage of direct consults. (B) Percentage of indirect consults. (DOCX) [file pone.0340735.s005.docx]

(A)


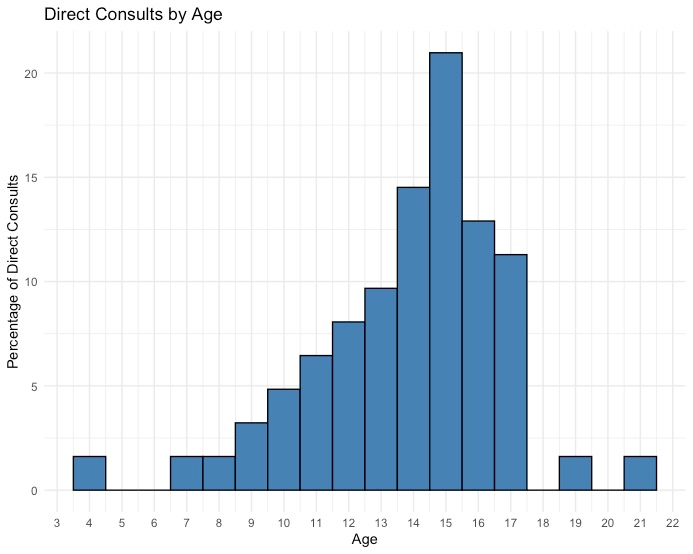


(B)


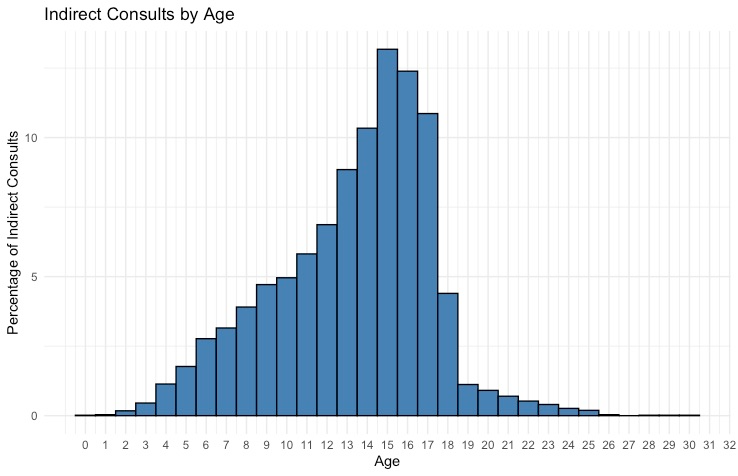


**Sup Fig 5. Age distribution of patient encounters by type of consult.** (A) Percentage of direct consults. (B) Percentage of indirect consults.
